# Urine Output Is Associated With In-hospital Mortality in Intensive Care Patients With Septic Shock: A Propensity Score Matching Analysis

**DOI:** 10.3389/fmed.2021.737654

**Published:** 2021-11-18

**Authors:** Tianyang Hu, Zhao Qiao, Ying Mei

**Affiliations:** ^1^Department of Cardiology, The Second Affiliated Hospital, Chongqing Medical University, Chongqing, China; ^2^Health Management Center, The Second Affiliated Hospital, Chongqing Medical University, Chongqing, China

**Keywords:** septic shock, urine output, MIMIC-IV, in-hospital mortality, propensity score matching

## Abstract

**Background:** The relationship between urine output (UO) and in-hospital mortality in intensive care patients with septic shock is currently inconclusive.

**Methods:** The baseline data, UO, and in-hospital prognosis of intensive care patients with septic shock were retrieved from the Medical Information Mart for Intensive Care IV (MIMIC-IV) database. By drawing receiver operating characteristic (ROC) curves and comparing the areas under the ROC curves (AUC) to determine the predictive value of UO for in-hospital mortality, and by drawing the Kaplan-Meier curves to compare the difference in in-hospital mortality between different groups of UO.

**Results:** Before and after the propensity score matching (PSM) analysis, UO was always a risk factor for in-hospital mortality in patients with septic shock. The AUC of UO was comparable to the Sequential Organ Failure Assessment (SOFA) scoring system, while the AUC of combining UO and SOFA was greater than that of SOFA. The median survival time of the high-UO group (UO > 0.39 ml/kg/h, before PSM; UO > 0.38 ml/kg/h, after PSM) was longer than that of the low-UO group. Compared with the high-UO group, the hazard ratios (HR) of the low-UO group were 2.6857 (before PSM) and 1.7879 (after PSM).

**Conclusions:** UO is an independent risk factor for septic shock. Low levels of UO significantly increase the in-hospital mortality of intensive care patients with septic shock. The predictive value of UO is comparable to the SOFA scoring system, and the combined predictive value of the two surpasses SOFA alone.

## Introduction

Sepsis is a life-threatening organ dysfunction caused by a dysregulated host response to infection. Septic shock is the most severe form of sepsis, which is characterized by persisting hypotension requiring vasopressors to maintain mean arterial pressure ≥65 mmHg and having an increased serum lactate level >2 mmol/L despite adequate volume resuscitation (1, 2). The in-hospital mortality rate of sepsis exceeds 10%, and septic shock is even worse (1, 3). A meta-analysis of European and North American populations showed that the in-hospital mortality rate of septic shock was as high as 39% (95% CI: 34.4–43.9%) (4). It is of far-reaching significance to clarify the independent risk factors related to mortality, which can further guide nursing and treatment, so as to achieve the purpose of reducing mortality especially in the intensive care unit (ICU).

Daily urine output (UO) is measured routinely in the ICU, and its prognostic value has already emerged. In 2013, Oh et al. (5) found that UO was significantly associated with the prognosis in critically ill patients with acute kidney injury (AKI) requiring continuous renal replacement therapy (CRRT). When the timing of CRRT initiation was stratified by 6 h UO, 28-day all-cause mortality rates were significantly lower in the non-oliguric group compared with the oliguric group. Huang et al. (6) found that reduced initial 24 h UO was associated with an increased risk in 7- and 30-day all-cause mortality and major adverse cardiovascular events (MACE) in ST-segment elevation myocardial infarction (STEMI) patients admitted without cardiogenic shock and renal dysfunction. Zhang et al. (7) investigated the relationship between UO on the first day of admission to the ICU and the in-hospital mortality of unselected critically ill patients and found that UO was an independent risk factor of mortality regardless of whether diuretics were used or not. Oliguria is one of the important signs of hypoperfusion in septic shock (8). However, due to the complexity of the composition of patients admitted to the ICU, it is not known whether the conclusion of Zhang et al. is applicable to septic shock. To date, no researches have confirmed the relationship between UO and mortality of patients with septic shock. This study is based on a well-known public database, Medical Information Mart for Intensive Care IV (MIMIC-IV) database, to investigate the relationship between UO on the first day of admission and the in-hospital mortality of intensive care patients with septic shock.

## Methods

### Database

MIMIC-IV (https://mimic.mit.edu/) builds upon the MIMIC-III database (9) and has made many improvements. MIMIC-IV contains comprehensive information (laboratory measurements, medications administered, vital signs documented, etc.) of patients admitted to a Tertiary Academic Medical Center in Boston, MA, USA between 2008 and 2019. The database is designed to support a wide variety of healthcare research. An individual who passed the “Protecting Human Research Participants” exam on the National Institutes of Health website can access the database (certification number 37474354 for author Tianyang Hu).

All patients in the database are anonymous and no informed consent is required.

### Study Population and Data Extraction

The patients diagnosed with “septic shock” in the MIMIC-IV database are divided into two categories: “septic shock” with International Classification of Disease (ICD) code 78552 (9th revision), and “severe sepsis with septic shock” with ICD code (10th revision). The inclusion criteria were: (1) aged≥18 years; (2) UO (ml/day) assessed within 24 h from admission; (3) UO (ml/kg/h) could be calculated within 24 h from admission. Post-procedural septic shock was excluded. Since the same patient may have multiple admission records, we only included the first ICU stay for each patient.

The following data was extracted from the MIMIC-IV database (version 1.0) by Navicat Premium software (version 15.0): age, gender, length of ICU stay, length of hospital stay, Charlson comorbidity index, Sequential Organ Failure Assessment (SOFA) score/ hemoglobin/white blood cells/platelets/creatinine/blood urea nitrogen/total bilirubin/heart rate/mean arterial pressure/respiratory rate/weight/urine output/ whether complicated with AKI/whether to take diuretics/whether treated with renal replacement therapy (RRT) on the first day of admission and hospital expire flag (a binary flag which indicates whether the patient died in hospital). Charlson comorbidity index (10) is a scoring system to quantify comorbidities (including myocardial infarct, congestive heart failure, peripheral vascular disease, cerebrovascular disease, dementia, chronic pulmonary disease, rheumatic disease, peptic ulcer disease, liver disease, diabetes, paraplegia, renal disease, malignant cancer, metastatic solid tumor, and acquired immunodeficiency syndrome). Diuretics mainly include bumetanide, chlorothiazide, furosemide, hydrochlorothiazide, metolazone, and spironolactone. If a variable was assessed multiple times on the first day of admission, took the average value.

### Statistical Analysis

Use the Kolmogorov-Smirnov test to evaluate whether the variables follow the normal distribution. If followed, then express the variable as mean ± standard deviation (M ± SD) and compare with independent sample *t*-test; if not follow, then express the variable as the median with interquartile range (IQR) and compare with Wilcoxon rank-sum test. Categorical variables were expressed as numbers and percentages, and compared by Chi-square test. Binomial Logistic regression analysis was conducted to evaluate the impact of UO on in-hospital mortality in patients with septic shock. Variables with a *P*-value <0.1 in the univariate analysis were included in the multivariate analysis. Z test was conducted following the method of Delong et al. (11) to compare the predictive value of UO, SOFA, and UO+SOFA by comparing the area under curves (AUC) of the receiver operating characteristic curves (ROC).

To reduce potential bias, propensity score matching (PSM) analysis was performed between the death group and survival group. All potential confounders were included in the PSM analysis: age, gender, Charlson comorbidity index, hemoglobin, white blood cells, platelets, creatinine, blood urea nitrogen, total bilirubin, heart rate, mean arterial pressure, respiratory rate on the first day of admission, whether complicated with AKI, whether to take diuretics, and whether treated with RRT on the first day of admission. The PSM analysis was performed by a 1:1 nearest neighbor matching algorithm (a caliper of 0.001) without replacement, and the propensity score was calculated by the logistic regression model.

In-hospital mortality is regarded as a time-to-event variable and the event is death during hospitalization. Patients were censored when they were discharged alive, and patients were followed during the hospital stay (7). UO was divided into high-UO group and low-UO group according to the optimal cut-off value indicated by the ROC curve, and Kaplan-Meier survival curves were drawn. The log-rank test was used to evaluate whether there was a difference in survival rate between the two groups.

All the analyses were performed using the software IBM SPSS Statistics (v26.0; IBM, Armonk, NY) and MedCalc (v19.6.1; MedCalc Software Ltd, Ostend, Belgium). A *P*-value < 0.05 was considered to be statistically significant.

## Results

### Baseline Characteristics

**Figure 1:**
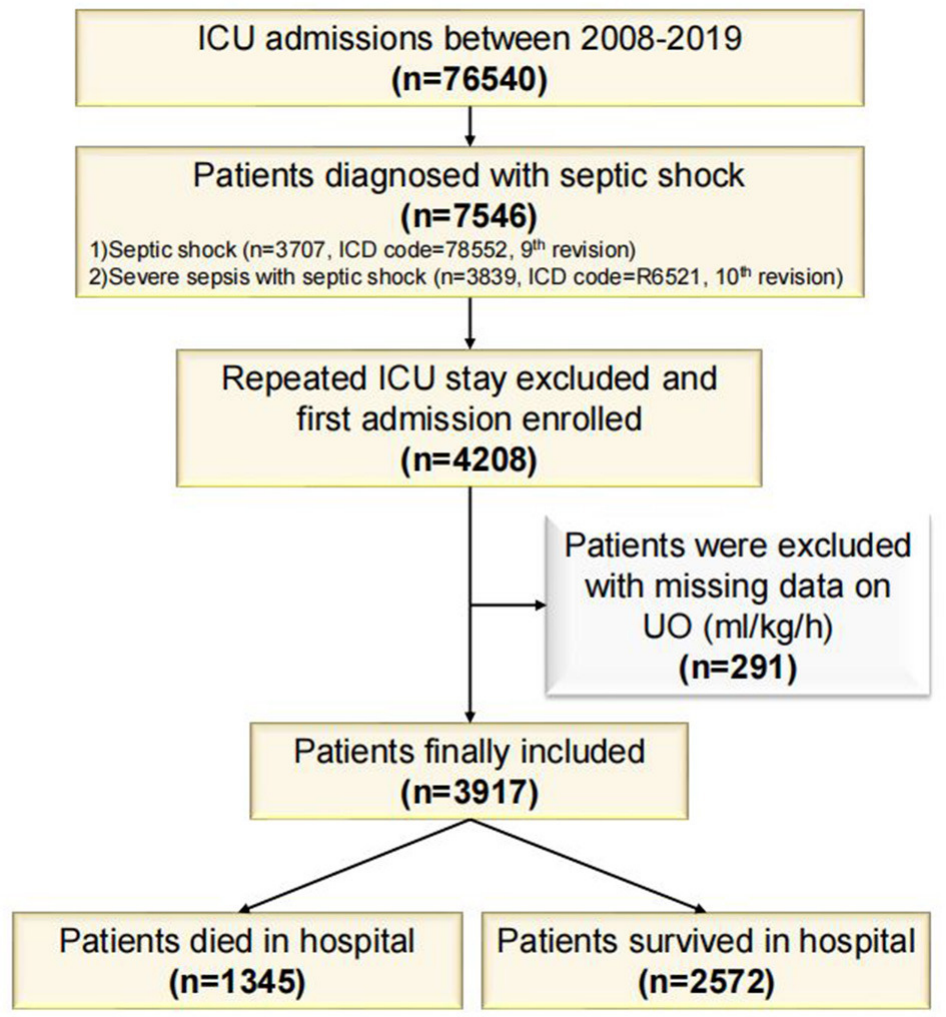
Flowchart of study cohort. ICU, Intensive Care Unit; ICD, International Classification of Disease; UO, Urine Output.

MIMIC-IV database contains 76,540 ICU admissions. Finally, 3,917 patients were included in this study (of which 1,345 died and 2,572 survived in the hospital, Figure 1) and the in-hospital mortality rate was 34.34%. The length of hospital stay of the survival group was longer than that of the death group (*P* < 0.001). Age, Charlson Comorbidity Index, SOFA score, the level of creatinine, total bilirubin, blood urea nitrogen, heart rate and respiratory rate on the first day of admission in the death group were higher than in the survival group significantly (*P* < 0.001 for all). Mean arterial pressure, the level of hemoglobin and platelets in the survival group were higher than in the death group significantly (*P* < 0.001 for all). UO in the survival group was higher than in the death group significantly (*P* < 0.001). The proportions of complicated with AKI, treated with diuretics and RRT in the death group were higher than in the survival group (*P* < 0.001 for all). After PSM with age, gender, Charlson comorbidity index, the level of hemoglobin, white blood cells, platelets, creatinine, blood urea nitrogen, total bilirubin, heart rate, mean arterial pressure, respiratory rate, whether complicated with AKI, whether to take diuretics, and whether treated with RRT on the first day of admission, the matching variables were balanced and comparable between the two groups (of which 963 died and 963 survived, *P* > 0.05 for all, Table 1). After PSM, the length of hospital stay of the survival group was still longer than that of the death group (*P* < 0.001). SOFA score on the first day of admission in the death group was higher than in the survival group significantly (*P* < 0.001), and UO in the survival group was still significantly higher than in the death group (*P* < 0.001). The baseline characteristics were presented in Table 1.

**Table 1:** Demographic and clinical characteristics of the study population.

	**Before PSM**			**After PSM**		
**Characteristics**	**Death (*n* = 2,572)**	**Survival (*n* = 1,345)**	***P***	**Death (*n* = 963)**	**Survival (*n* = 963)**	***P***
^*^Age, year	71 (60–82)	68 (56–79)	0.000	72 (61–82)	72 (61–82)	0.961
^*^Gender, male	723 (53.8)	1398 (54.4)	0.720	521 (54.1)	512 (53.1)	0.681
LOS hospital, day	6.5 (2.0–14.5)	11.5 (6.6–20.6)	0.000	7.1 (2.5–14.6)	12.7 (7.1–21.6)	0.000
LOS ICU, day	3.7 (1.5–8.7)	3.4 (1.9–7.7)	0.243	3.9 (1.6–8.6)	3.8 (2.1–9.0)	0.005
^*^CCI	7 (5–9)	6 (4–8)	0.000	7 (5–9)	7 (5–9)	0.869
Laboratory tests						
^*^Hb, g/dL	9.8 (8.5–11.4)	10.3 (8.9–11.7)	0.000	9.9 (8.6–11.5)	10.2 (8.8–11.5)	0.147
^*^WBC, 10^9^/L	14.0 (8.2–20.3)	13.8 (9.3–19.3)	0.854	14.1 (8.2–20.4)	13.7 (9.1–20.3)	0.776
^*^PLT, 10^9^/L	156 (89–241)	183 (124–255)	0.000	169 (99–259)	163 (115–236)	0.668
^*^Cr, ng/dL	1.7 (1.2–2.7)	1.3 (0.9–2.0)	0.000	1.5 (1.1–2.5)	1.5 (1.0–2.4)	0.617
^*^BUN, mmol/L	36.0 (23.5–56.0)	26.0 (16.5–42.3)	0.000	33.5 (22.0–52.0)	32.5 (20.5–52.5)	0.318
^*^TBil, mg/dL	1.6 (0.6–2.9)	1.1 (0.5–2.7)	0.000	1.4 (0.6–2.7)	1.3 (0.6–2.7)	0.778
Vital signs						
^*^HR, bpm	96 (82–108)	90 (78–102)	0.000	94 (80–107)	94 (81–106)	0.899
^*^MAP, mmHg	71 (65–76)	72 (68–77)	0.000	71 (66–77)	72 (67–77)	0.207
^*^RR, cpm	22 (19–26)	21 (18–24)	0.000	22 (19–25)	22 (19–25)	0.519
SOFA score	12 (9–15)	8 (5–11)	0.000	11 (8–14)	8 (6–12)	0.000
Day 1 UO, ml/day	595 (186–1264)	1400 (825–2317)	0.000	715 (264–1400)	1170 (649–1950)	0.000
Day 1 UO, ml/kg/h	0.32 (0.10–0.67)	0.75 (0.41–1.25)	0.000	0.38 (0.13–0.73)	0.63 (0.33–1.05)	0.000
^*^Day 1 AKI	425 (31.6)	538 (20.9)	0.000	264 (27.4)	254 (26.3)	0.607
^*^Day 1 diuretic	269 (20.0)	318 (12.4)	0.000	166 (17.2)	161 (16.7)	0.762
^*^Day 1 RRT	366 (27.2)	271 (10.5)	0.000	181 (18.8)	182 (18.9)	0.954

*Values are expressed as the median (IQR) or n (%)*.

*PSM, Propensity Score Matching; LOS, Length of Stay; ICU, Intensive Care Unit; CCI, Charlson Comorbidity Index; Hb, Hemoglobin; WBC, White Blood Cells; PLT, Platelets; Cr, Creatinine; BUN, Blood Urea Nitrogen; TBil, Total Bilirubin; HR, Heart Rate; bpm, beat per minute; MAP, Mean Arterial Pressure; RR, Respiratory Rate; cpm, count per minute; SOFA, Sequential Organ Failure Assessment; UO, Urine Output; AKI, Acute Kidney Injury; RRT, Renal Replacement Therapy*.

* *Covariables included in the PSM*.

### Logistic Regression Analysis

Considering the collinearity between UO (ml/day) and UO (ml/kg/h), the latter was included in the binomial logistic regression analysis. UO was a risk factor for in-hospital mortality in patients with septic shock before (OR: 0.285, 95% CI: 0.247–0.330, *P* < 0.001) and after (OR: 0.507, 95% CI: 0.434–0.593, *P* < 0.001) adjustment (Table 2). After PSM, UO was still a risk factor for in-hospital mortality in patients with septic shock before (OR: 0.544, 95% CI: 0.465–0.638, *P* < 0.001) and after (OR: 0.678, 95% CI: 0.578–0.796, *P* < 0.001) adjustment (Table 3).

**Table 2:** Binomial Logistic regression analysis of urine output for in-hospital mortality among intensive care patients with septic shock (before PSM).

**Variable**	**Univariable**		**Multivariable**	
	**OR (95% CI)**	***P***	**OR (95% CI)**	***P***
Age	1.014 (1.010–1.018)	0.000	1.009 (1.002–1.016)	0.007
Gender (male)	0.976 (0.855–1.114)	0.720		
LOS hospital	0.976 (0.971–0.982)	0.000	0.966 (0.960–0.973)	0.000
LOS ICU	1.004 (0.996–1.013)	0.282		
CCI	1.161 (1.133–1.188)	0.000	1.143 (1.107–1.181)	0.000
Hemoglobin	0.925 (0.894–0.956)	0.000	0.942 (0.904–0.982)	0.005
WBC	1.005 (0.999–1.010)	0.088	0.998 (0.992–1.004)	0.532
Platelets	0.998 (0.998–0.999)	0.000	1.001 (1.000–1.001)	0.095
Creatinine	1.182 (1.131–1.234)	0.000	0.719 (0.661–0.781)	0.000
BUN	1.015 (1.012–1.017)	0.000	1.009 (1.005–1.014)	0.000
Total bilirubin	1.182 (1.131–1.234)	0.000	1.035 (1.017–1.054)	0.000
Heart rate	1.016 (1.013–1.020)	0.000	1.017 (1.012–1.022)	0.000
MAP	0.967 (0.959–0.975)	0.000	0.988 (0.979–0.998)	0.016
Respiratory rate	1.086 (1.069–1.102)	0.000	1.045 (1.025–1.066)	0.000
SOFA score	1.015 (1.012–1.017)	0.000	1.175 (1.147–1.203)	0.000
Day 1 UO, mg/kg/h	0.285 (0.247–0.330)	0.000	0.507 (0.434–0.593)	0.000
Day 1 AKI	1.747 (1.505–2.027)	0.000	1.030 (0.854–1.240)	0.760
Day 1 diuretic	1.772 (1.483–2.117)	0.000	1.590 (1.284–1.968)	0.000
Day 1 RRT	3.174 (2.667–3.777)	0.000	2.285 (1.779–2.935)	0.000

*PSM, Propensity Score Matching; OR, Odds Ratio; CI, Confidence Interval; LOS, Length of Stay; ICU, Intensive Care Unit; CCI, Charlson Comorbidity Index; WBC, White Blood Cells; BUN, Blood Urea Nitrogen; MAP, Mean Arterial Pressure; SOFA, Sequential Organ Failure Assessment; UO, Urine Output; AKI, Acute Kidney Injury; RRT, Renal Replacement Therapy*.

**Table 3:** Binomial Logistic regression analysis of urine output for in-hospital mortality among intensive care patients with septic shock (after PSM).

**Variable**	**Univariable**		**Multivariable**	
	**OR (95% CI)**	***P***	**OR (95% CI)**	***P***
Age	1.001 (0.995–1.007)	0.858		
Gender, male	1.038 (0.868–1.242)	0.681		
LOS hospital	0.981 (0.974–0.987)	0.000	0.979 (0.972–0.986)	0.000
LOS ICU	0.992 (0.982–1.002)	0.106		
CCI	1.006 (0.974–1.039)	0.715		
Hemoglobin	0.982 (0.939–1.027)	0.434		
WBC	0.999 (0.992–1.006)	0.792		
Platelets	1.001 (1.000–1.001)	0.099	1.002 (1.001–1.002)	0.000
Creatinine	1.005 (0.948–1.066)	0.871		
BUN	1.001 (0.998–1.004)	0.601		
Total bilirubin	0.999 (0.979–1.020)	0.913		
Heart rate	1.000 (0.994–1.005)	0.853		
MAP	0.999 (0.989–1.009)	0.801		
Respiratory Rate	0.993 (0.973–1.014)	0.513		
SOFA score	1.102 (1.078–1.127)	0.000	1.105 (1.079–1.133)	0.000
Day 1 UO, mg/kg/h	0.544 (0.465–0.638)	0.000	0.678 (0.578–0.796)	0.000
Day 1 AKI	1.054 (0.862–1.290)	0.607		
Day 1 diuretic	1.038 (0.818–1.316)	0.762		
Day 1 RRT	0.993 (0.790–1.248)	0.954		

*PSM, Propensity Score Matching; OR, Odds Ratio; CI, Confidence Interval; LOS, Length of Stay; ICU, Intensive Care Unit; CCI, Charlson Comorbidity Index; WBC, White Blood Cells; BUN, Blood Urea Nitrogen; MAP, Mean Arterial Pressure; SOFA, Sequential Organ Failure Assessment; UO, Urine Output; AKI, Acute Kidney Injury; RRT, Renal Replacement Therapy*.

### Comparison of ROC Curves

**Figure 2:**
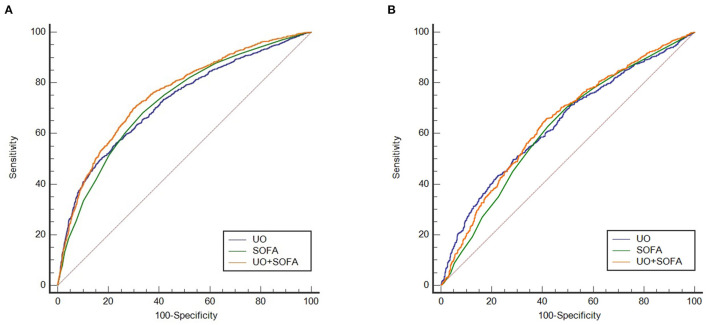
**(A)** ROC curves of UO, SOFA, UO + SOFA (before propensity score matching); **(B)** ROC curves of UO, SOFA, UO + SOFA (after propensity score matching). UO, Urine Output (mg/kg/h); SOFA, Sequential Organ Failure Assessment.

The ROC curves were drawn to clarify the predictive value of UO for in-hospital mortality of septic shock (Figure 2). Before PSM (Figure 2A), the AUCs of UO, SOFA, combining UO and SOFA (UO + SOFA) were 0.722, 0.725, and 0.753, respectively (Table 4). The AUC of UO was comparable to SOFA (*Z* = 0.237, *P* = 0.8127), while the AUC of UO+SOFA was greater than that of UO (*Z* = 5.079, *P* < 0.0001) and SOFA (*Z* = 6.264, *P* < 0.0001). UO+SOFA had the highest sensitivity (69.81%) and Youden's index (0.4007), while UO had the highest specificity (76.94%). After PSM (Figure 2B), the AUCs of UO, SOFA, and UO+SOFA were 0.637, 0.622, and 0.643, respectively (Table 5). The AUC of UO was still comparable to SOFA (*Z* = 1.090, *P* = 0.2756), while the AUC of UO + SOFA was also greater than that of SOFA (*Z* = 3.034, *P* = 0.0024), but comparable to UO (*Z* = 0.640, *P* = 0.5219). UO+SOFA had the Youden's index (0.2420), while UO had the highest specificity (71.55%) and SOFA had the highest sensitivity (70.40%).

**Table 4:** Comparison of ROC curves (before PSM).

**Factor**	**AUC**	**95%CI**	**Optimal cut-off**	**Sensitivity**	**Specificity**	**Youden's index**
UO	0.722	0.708~0.736	0.39	56.21	76.94	0.3315
SOFA	0.725	0.710~0.739	9	68.18	66.45	0.3462
UO + SOFA	0.753	0.740~0.767	0.34265^*^	69.81	70.26	0.4007

*UO, Urine Output (mg/kg/h); SOFA, Sequential Organ Failure Assessment*.

* *Prediction probability of logistic regression model for combining UO and SOFA, corresponding to UO = 0.94, SOFA = 11*.

**Table 5:** Comparison of ROC curves (after PSM).

**Factor**	**AUC**	**95%CI**	**Optimal cut-off**	**Sensitivity**	**Specificity**	**Youden's index**
UO	0.637	0.615~0.659	0.38	49.64	71.55	0.2118
SOFA	0.622	0.600~0.644	8	70.40	50.36	0.2077
UO + SOFA	0.643	0.621~0.664	0.49034^*^	65.84	58.36	0.2420

*UO, Urine Output (mg/kg/h); SOFA, Sequential Organ Failure Assessment*.

* *Prediction probability of logistic regression model for combining UO and SOFA, corresponding to UO = 0.58, SOFA = 9*.

### Comparison of Kaplan-Meier Curves

**Figure 3:**
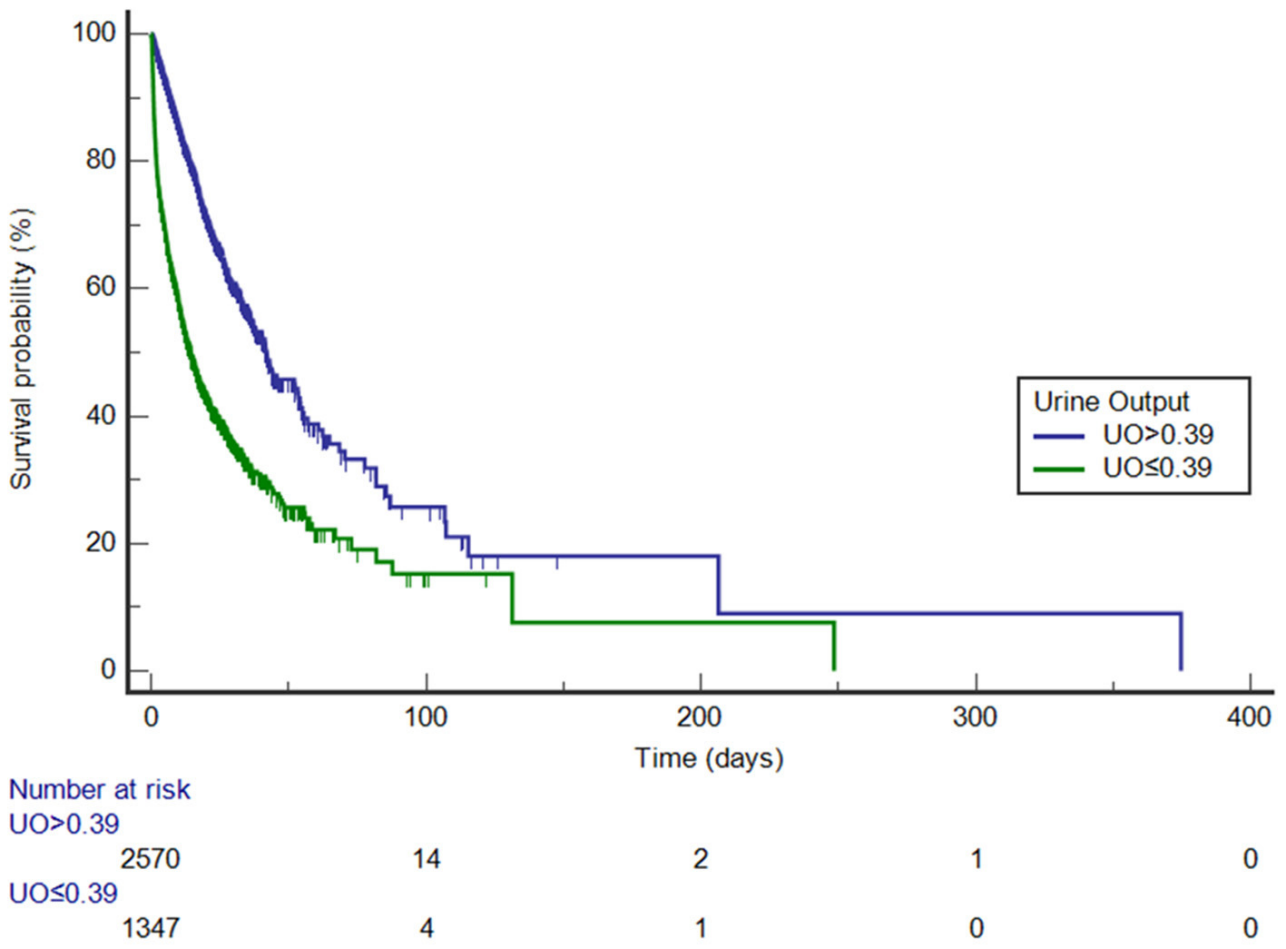
Kaplan-Meier survival curves by urine output category (before propensity score matching, log-rank *P* < 0.0001). UO, Urine Output (mg/kg/h).

**Figure 4:**
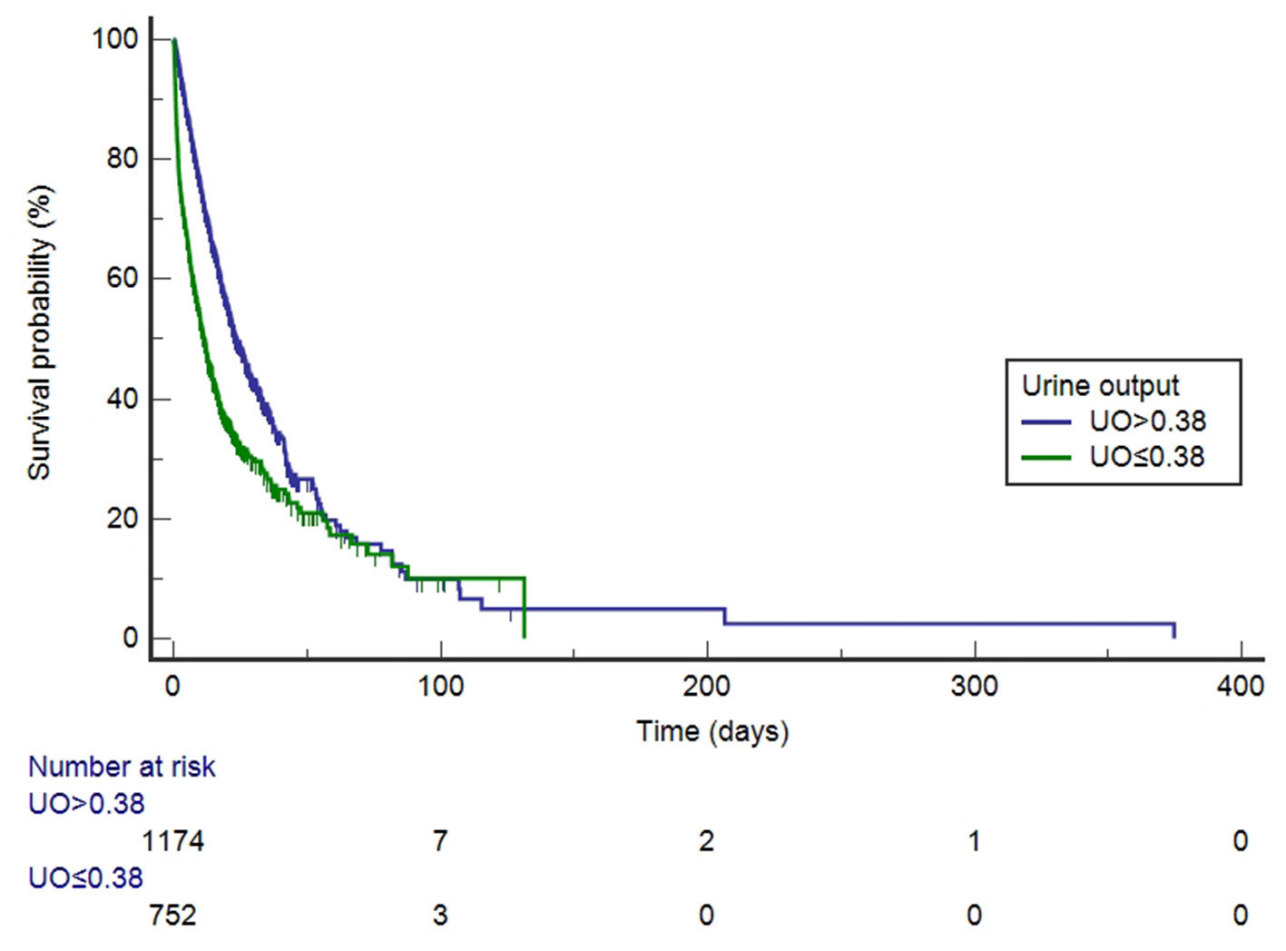
Kaplan-Meier survival curves by urine output category (after propensity score matching, log-rank *P* < 0.0001). UO, Urine Output (mg/kg/h).

Before PSM, UO was divided into high-UO group and low-UO group with the optimal cut-off value of 0.39 ml/kg/h. The Kaplan-Meier curves are shown in Figure 3. The median survival time of the high-UO group was 42.097 days (95% CI: 37.842–52.060), while of the low-UO group was 14.470 days (95% CI: 12.726–16.674), and the difference was statistically significant (*P* < 0.0001). Compared with the high-UO group, the hazard ratio (HR) of the low-UO group was 2.6857 (95% CI: 2.3955–3.0112). After PSM, the optimal cut-off value was 0.38 ml/kg/h (Figure 4). The median survival time of the high-UO group was 23.632 days (95% CI: 21.448–27.116), while of the low-UO group was 11.449 days (95% CI: 9.955–12.926), and the difference was statistically significant (*P* < 0.0001). Compared with the high-UO group, the HR of the low-UO group was 1.7879 (95% CI: 1.5669–2.0401).

## Discussion

To the best of our knowledge, this study investigated the relationship between urine output on the first day of admission and in-hospital mortality of intensive care patients with septic shock for the first time. We confirmed that UO is an independent risk factor for septic shock. Before PSM, the optimal cut-off value determined by the ROC curve was 0.39 mg/kg/h. Compared with the high-UO group, the HR of the low-UO group was 2.6857, suggesting that the risk of in-hospital death in the low-UO group was 2.6857 times that of the high-UO group. The optimal cut-off value after PSM was 0.38 mg/kg/h, which was almost the same as before PSM. It was found that the risk of in-hospital death in the low-UO group was 1.7879 times that of the high-UO group. Therefore, the above results indicate that a low level of UO on the first day is significantly associated with an increase in in-hospital mortality.

The PSM is a “post-randomization” statistical analysis method, which reduces the influence of biases and confounding variables on the results in retrospective studies to a certain extent. AKI is the most frequent complication in septic shock and RRT is the standard of care for severe AKI (12). If patients with septic shock have oliguria or anuria on admission, they may progress to AKI at a later stage. A study showed that 3–5 h consecutive oliguria in patients with septic shock may be an indicator to measure the risk of AKI (13). Meanwhile, the most frequent indication for acute dialysis was oliguria (14). In general, whether patients are complicated with AKI and whether to undergo RRT or diuretic therapy are significantly related to UO theoretically. We have also balanced some important laboratory tests and vital signs. Among them, white blood cells count is related to infection and is often used to detect sepsis (15); creatinine and BUN can reflect renal function (16); platelets and total bilirubin can reflect coagulation function and liver function (17); respiratory rate is related to respiratory function; hemoglobin not only reflects the presence of anemia, but also is related to oxygenation (18). We were trying to evaluate indicators related to cardiac function, but the missing values of indicators such as troponin and BNP in the MIMIC-IV database are too many, and some even exceed 90%. Imaging data of cardiac function, such as ejection fraction, are currently not available in MIMIC-IV database. Thus, we finally used heart rate and mean arterial pressure as matching parameters to minimize the cardiac function bias between the two groups. After PSM, although the risk of in-hospital death in the low-UO group was lower than before, it still had approximately twice the risk of death (1.7879 times) compared to the high-UO group. It can be seen that the UO on the first day of admission does not depend on whether complicated with AKI and whether to undergo RRT or diuretic therapy, but is directly related to the prognosis of the patients, reflecting the independence of its predictive value.

Septic shock is a complex syndrome with severe hemodynamic changes, manifested by profound cardiovascular derangements, redistribution of blood flow between organs, and microcirculatory alterations (2). Cardiovascular derangements and the redistribution of blood flow can seriously affect splanchnic circulation, and if the kidney is compromised, it will correspondingly lead to a decrease in UO. Meanwhile, sepsis, especially in severe patients, is almost invariably related to altered coagulation, which can easily lead to disseminated intravascular coagulation (19) and microvascular thrombosis (20). Thrombus formation leads to insufficient tissue perfusion. Since the septic shock is a subtype and severe type of sepsis, which is more prone to progress to coagulation dysfunction and eventually leads to insufficient tissue perfusion, resulting a decrease in UO. In addition, septic shock can cause a vasodilatory state due to excessive NO production, vasopressin deficiency and resistance (21, 22), and further aggravate tissue hypoperfusion. The above reasons are independent of AKI and lead a decrease in UO, therefore, even after adjusting for confounding factors such as AKI, UO is still an independent risk factor for in-hospital mortality in patients with septic shock.

The essence of sepsis and septic shock is organ dysfunction, and the severity of organ dysfunction has been assessed through various scoring systems. Currently, SOFA is the predominant scoring system used for sepsis and septic shock, and it is also one of the definitions of sepsis recognized by Sepsis-3 (1). Several studies have confirmed the value of SOFA in predicting the mortality of septic shock (23–28), but its performance is not satisfactory. In our study, before PSM, the AUC of UO exceeded 0.7, which was of moderate predictive value. After PSM, AUC dropped to 0.637, suggesting that the predictive value was limited. However, the predictive value of UO was always comparable to the SOFA score system. Even so, the application of UO alone in the prediction of in-hospital mortality for septic shock still lacks practical significance. The combination of predictors may improve prediction performance. As the task force of Sepsis-3 pointed out, there are many novel biomarkers that can identify renal dysfunction or coagulopathy earlier than the elements used in SOFA, but they need to be more extensively verified before they are incorporated into the clinical criteria for sepsis (1). In this study, the combined prediction efficiency of UO and SOFA was higher than that of SOFA alone. However, since the respective weights were not given, the corresponding cut-off values of the two were not suitable for predicting in-hospital mortality directly. Thus, it may be a better choice to consider creating a new scoring system, such as incorporating UO into SOFA as a factor, similar to the “UO-corrected SOFA scoring system.”

Our findings emphasize the importance of monitoring UO in clinical practice in order to identify high-risk patients with septic shock early and intervene as soon as possible to achieve the goal of reducing in-hospital mortality. UO monitoring is easy to perform and inexpensive, and is especially suitable for promotion in countries with limited resources. We recommend using the weight-corrected UO, namely UO (ml/kg/h). Moreover, we also emphasize the importance of UO in the hemodynamic management of septic shock. Hemodynamic support for patients with septic shock is crucial (29), including the use of large amounts of fluids in combination with vasopressors, and in some cases with inotropic agents. The hemodynamic targets for resuscitation of septic shock often rely on macro-hemodynamic parameters, including heart rate, mean arterial pressure, and central venous pressure. However, despite the restoration of macro-hemodynamic parameters, persistent alterations in microcirculatory blood flow can still lead to organ failure (2, 30, 31). This dissociation between the macrocirculation and microcirculation is the so-called “a loss of hemodynamic coherence” (32). UO reflects renal perfusion and is also an effective indicator of microcirculation perfusion. Thus, monitoring UO may play a positive role in hemodynamic management for septic shock.

We must point out the limitations of this study: (1) The patients in the MIMIC-IV database are mainly white, and a large number of patients cannot be identified by ethnicity. Therefore, the variable ethnicity was not included in the PSM analysis, which has a potential impact on the results; (2) At present, it is difficult to identify the exact sites of infection and causative organisms of the patients in the database. The predictive value of UO for septic shock caused by different reasons or in different sites of infection (such as kidney vs. other sites) may be significantly different; (3) The daily fluid intake (including drinking water) of the patients will also affect the UO, but since the exact values of these variables cannot be obtained, the influence of these confounding factors on the results cannot be ignored. It should be pointed out that a large part of the patients in this study may not have been diagnosed with septic shock at the time of admission. Therefore, our findings may also be applicable to intensive care patients with the potential to develop septic shock. The advantage of this study lies in the large sample size, which allows us to have enough space for the PSM analysis and makes our conclusions more reliable.

## Conclusions

UO is an independent risk factor for septic shock. Low levels of UO significantly increase the in-hospital mortality of intensive care patients with septic shock. The predictive value of UO for patients with septic shock is comparable to the SOFA scoring system, and the combined predictive value of the two surpasses SOFA alone. Since the above results are based on this retrospective study, rigorous prospective clinical trials are still needed to confirm.

## Data Availability Statement

The raw data supporting the conclusions of this article will be made available by the authors, without undue reservation.

## Ethics Statement

Ethical review and approval was not required for the study on human participants in accordance with the local legislation and institutional requirements. Written informed consent for participation was not required for this study in accordance with the national legislation and the institutional requirements.

## Author Contributions

TH and YM conceived and designed the study. TH and ZQ extracted the data, analyzed and interpreted the data, and drafted the work. YM participated in design of the study, assisted with revisions of the manuscript, and takes responsibility for the content of the manuscript including the data and analysis. All authors have approved the final version of the manuscript for submission and agree to be accountable for all aspects of the work.

## Conflict of Interest

The authors declare that the research was conducted in the absence of any commercial or financial relationships that could be construed as a potential conflict of interest.

## Publisher's Note

All claims expressed in this article are solely those of the authors and do not necessarily represent those of their affiliated organizations, or those of the publisher, the editors and the reviewers. Any product that may be evaluated in this article, or claim that may be made by its manufacturer, is not guaranteed or endorsed by the publisher.

## Abbreviations

UO: urine output
MIMIC-IV: Medical Information Mart for Intensive Care IV
AKI: acute kidney injury
CRRT: continuous renal replacement therapy
MACE: major adverse cardiovascular events
STEMI: ST-segment elevation myocardial infarction
ROC: receiver operating characteristic
AUC: areas under the receiver operating characteristic curves
PSM: propensity score matching
SOFA: Sequential Organ Failure Assessment
HR: hazard ratio
OR: Odds Ratio
CI: Confidence Interval
ICU: intensive care unit
ICD: International Classification of Disease
RRT: renal replacement therapy
M ± SD: mean ± standard deviation
IQR: interquartile range
LOS: Length of Stay
CCI: Charlson Comorbidity Index
Hb: Hemoglobin
WBC: White Blood Cells
PLT: Platelets
Cr: Creatinine
BUN: Blood Urea Nitrogen
TBil: Total Bilirubin
HR: Heart Rate
bpm: beat per minute
MAP: Mean Arterial Pressure
RR: Respiratory Rate
cpm: count per minute.
